# Becoming More Resilient during COVID-19: Insights from a Process Evaluation of Digital Resilience Training

**DOI:** 10.3390/ijerph191912899

**Published:** 2022-10-08

**Authors:** Wei How Darryl Ang, Han Shi Jocelyn Chew, Yew Hui Nicholas Ong, Zhongjia James Zheng, Shefaly Shorey, Ying Lau

**Affiliations:** 1 Alice Lee Centre for Nursing Studies, Yong Loo Lin School of Medicine, National University of Singapore, Singapore 119077, Singapore; 2 Ministry of Education, Singapore 138675, Singapore

**Keywords:** resilience, randomized controlled trial, university students, process evaluation, qualitative

## Abstract

Resilience training is gaining attention as a strategy to build students’ resistance to adversity and promote their mental well-being. However, owing to inconsistencies and variations in the content and delivery of resilience training, more work is needed to examine students’ experiences and preferences to address issues relating to intervention fidelity. This study adopted a qualitative approach in exploring students’ experience of synchronous and asynchronous versions of a digital resilience training program. Seventeen students were interviewed using a semi-structured virtual face-to-face interview via Zoom. The thematic analyses unveiled four themes: embarking on a journey toward resilience, discovering strategies to develop resilience, finding a balance to benefit from resilience skill enhancement, and instilling resilience in the everyday. Future resilience training should consider students’ workload and interactivity to enhance their engagement. As being resilient is associated with better mental well-being, the findings of this study may support the development of future wellness programs.

## 1. Introduction

Undergraduate students experience a plethora of challenges such as managing academic demands during their time in university [1,2]. Unlike in pre-tertiary education, there are changes in the university environment, such as the complexity and vastness of the undergraduate curriculum and inadequate resources (e.g., facilities) [3,4]. These challenges are amplified due to the onset and persistence of the global coronavirus pandemic (COVID-19) [5,6]. First, the need for social distancing and isolation meant that students have to learn through an online platform [7,8] Second, learning over an online platform can pose challenges for students [8]. Owing to the sudden emergence of the COVID-19 pandemic, students struggled with a lack of basic online learning tools such as access to the Internet and devices that could support their learning [9]. For these reasons, the deleterious effects arising from these challenges may lead to greater levels of stress, potentially leading to negative downstream impacts resulting in poorer mental health [10,11].

Addressing concerns relating to mental health is important as the global prevalence rates for anxiety disorders extend from 28% to 33.8% [12,13] and depression ranges from 20% to 34% [14,15]. Mental health issues can lead to far-reaching adverse consequences such as higher risks of suicide [16], poorer academic outcomes [17], and inability to complete school [18]. These consequences are worrying for several stakeholders, educators, families, and university administrators. Notwithstanding, the rising prevalence of mental health disorders can lead to a greater burden on the health care systems [19,20]. Considering the enormous economic and psychological impacts of mental health illnesses, shifting focus from a treatment-oriented to a preventive stance is important to ensure that students do not become encumbered by mental health issues.

### 1.1. Theoretical Underpinning of Resilience

Resilience has been widely reported to have positive influences on students’ mental health and well-being [21,22,23]. The concept of resilience can be understood from a trait, process, or outcome perspective [24]. As a trait, being resilient refers to the presence of individual characteristics that promote an individual’s ability to recover from adversity [25]. These individual characteristics may arguably be inherited, due to genetics or are considered as a form of an acquired skill [26]. For example, it has been proposed that an individual with the ability to regulate emotion is considered to be resilient [25,26].

From a process perspective, Masten [27] defined resilience as “the manifested capacity of a dynamic system to adapt successfully to disturbances that threaten the function, survival or development of the system” (p. 187). The process orientation of resilience proposes that interactions between an individual and his or her environment can influence their resilience [27]. Conversely, as an outcome, it focuses on an individual’s state of being resilient in the face of adversity [28], without acknowledging the potential mechanisms that take place. Independently, the trait, process, and outcome definitions are limited as they do not consider how an individual’s trait, or potential mechanisms influence an individual’s resilience [29].

Through the salutogenic discourse, an individual’s health outcome is influenced by various factors and based on the individual’s interactions with the environment [29]. This highlights the importance of appreciating the trait, and mechanisms of resilience (process) in inoculating certain groups of people against the negative effects (outcome) of experienced adversities [30]. For these reasons, it will be important to examine resilience from a combination of trait, process, and outcome perspectives.

Therefore, this study defines resilience using an all-encompassing approach consisting of the trait, process, and outcome orientations. Resilience is defined by Van Breda [30] as “the multilevel processes that systems engage in to obtain better-than-expected outcomes in the face or wake of adversity” (p. 4). The part on multilevel processes suggests that resilience can be developed through various mechanisms [30]. A better-than-expected outcome represents how an individual institutes a positive outcome, which is influenced by his or her community or culture [30,31]. Moreover, for one to become resilient, several factors, ranging from personal to societal domains, are proposed to potentially play a resilience-enhancing role [32,33].

Existing resilience training among students has largely focused on the individual and interpersonal factors of promoting resilience [21,22,34]. However, a recent systematic review has concluded that although effective in improving students’ resilience and mental well-being, existing resilience training continues to be confounded by various factors such as different contents and features [21,35]. This conclusion may be attributed to several reasons.

First, it may be due to the adaptation of a different theoretical stance of resilience (e.g., trait, process, or outcome). Second, resilience training may have been conducted to address a variety of issues that is unique to a specific context or population. For instance, the majority of resilience training has been found to be conducted among certain groups of individuals based on their memberships (e.g., students) within the Western context [21,36]. Third, although the mechanism of resilience has been reported based on resilience theories [30,32], if these protective factors (e.g., personal, relational, or environmental) work in synergy or how students make sense of them through the training remains unclear. For these reasons, solely relying on the findings of meta-analyses of randomized controlled trials (RCTs) can be challenging as the design does not provide an in-depth understanding of the intricacies of how the intervention has worked. RCTs provide answers for the “does it work” angle, but they are unable to show how treatment fidelity, quality of the intervention, causal mechanisms, and presence of contextual factors account for any variation in the outcomes [37,38]. The integration of a process evaluation framework allows researchers to draw in-depth conclusions on how the intervention has affected its participants [37].

### 1.2. Theoretical Framework

**Figure 1:**
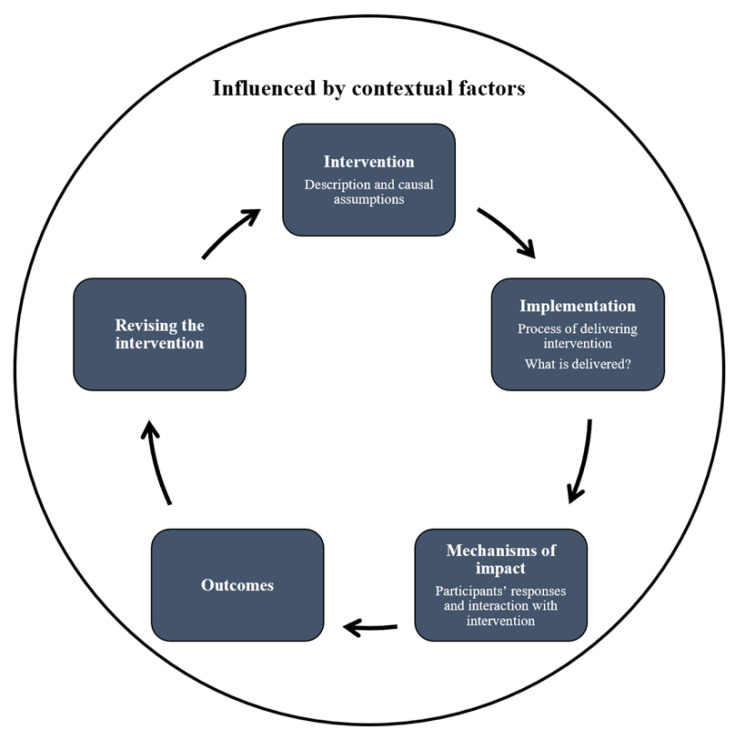
Process evaluation framework (Moore et al., 2014).

The Medical Research Council’s process evaluation framework (Figure 1) proposes three areas for researchers to evaluate: (1) intervention implementation, (2) causal mechanisms, and (3) contextual factors [37]. Evaluating the implementation of an intervention requires an examination of its quantity and quality [37]. This process involves answering a series of questions ranging from the “how” and “what.” It refers to the understanding of how the intervention (e.g., method of delivery) and what (e.g., the content) was delivered [38,39]. This leads to the second area that examines the mechanism of the intervention, which is important to allow further works to replicate a similar intervention [40]. Understanding the causal mechanisms of intervention goes beyond the assessment of effectiveness but requires a deeper exploration of how theorized pathway leads to an outcome [41]. Finally, examining how the context may affect the implementation is equally important. As context refers to anything external to the intervention, it may vary across settings. For instance, resilience is expressed differently in the East and the West. Individuals in the East view resilient behaviors as those adding value to the community, whereas Western cultures adopt a more individualistic approach [42,43].

To the authors’ knowledge, few studies have evaluated the effects of resilience training among students from a process evaluation angle. Contemporary undergraduate students are largely from Generation Z [44]. Considering that they possess different learning preferences from previous generations [44,45], soliciting their experiences to address questions relating to treatment fidelity is meaningful. Regarding the contents, a multitude of strategies are used to enhance resilience [21,36], and a closer examination of the effective strategies and their mechanisms is necessary. From an intervention design perspective, considerable gaps ranging from duration, frequency, and platform must be addressed [21,35,46]. With these considerations, process evaluations are carried out to provide deeper insights from the recipients’ perspective to understand how future trials may be refined.

Given the vastness of the information required for a process evaluation, a qualitative approach is appropriate to gather participants’ insights into how an intervention has influenced them [38]. Therefore, this study aimed to use a qualitative process evaluation approach to examine how students interacted with the asynchronous and synchronous versions of the digital resilience skill enhancement (RISE) program. The research questions were as follows:

1. What were students’ experiences of using the RISE program?
2. What were the contextual factors and mechanisms of the RISE program that influenced students’ resilience?
3. How did the RISE program impact students’ resilience?

## 2. Methods

### 2.1. Study Design

This study was part of an RCT that examined the effectiveness of the RISE program. The RCT was prospectively registered on the ClinicalTrials.gov registry (NCT05072340). To examine participants’ experiences, a descriptive qualitative design was used, and individual semi-structured interviews were conducted via Zoom. This study was reported in connection with the Consolidated Criteria for Reporting Qualitative Research Checklist [47].

### 2.2. Setting and Sample

This study was conducted in an autonomous public university in Singapore. The university enrolls students in undergraduate and postgraduate programs across 17 faculties and schools. Participants were eligible to participate in this qualitative study if they were: (1) aged above 18 years old, (2) pursuing any full-time undergraduate program, (3) able to comprehend the English language, (4) did not have any self-reported history of mental health disorders, and (5) completed the RISE program.

### 2.3. RISE Program

The RISE program was designed using a three-step approach concerning the Medical Research Council framework for developing complex interventions [48]. First, resilience theory [32] and the transactional model of stress and coping [49] provided the theoretical basis, which contributed to the mechanism of the contents used in the RISE program.

Second, systematic reviews, comprising two meta-analyses [21,35] and one meta-ethnography [23], were conducted to generate evidence. The reviews positioned resilience training as a promising strategy to foster students’ resilience. The reviews also contributed to the development of resilience-enhancing strategies and design considerations for the RISE program.

Finally, as part of the developmental process [48], a qualitative study using a user-centered approach was conducted to provide the contextual information for RISE [50]. The contextual information derived included students’ suggestions for resilience-enhancing strategies and preference for receiving resilience training. Based on the findings from the intervention development, the RISE program was designed as a six-week training delivered over the university’s online learning platform LumiNUS and synchronous communication software Zoom. The online learning platform was available in an application for any devices (e.g., mobile phones, tablets, or laptops) that can be connected to the Internet. Each week featured a different topic relating to building students’ resilience.

An RCT design was used to evaluate the effectiveness of the RISE program. The trial was conducted from December 2021 to January 2022 and recruited an eventual sample of 115 undergraduate students. The trial comprised two experimental groups, with 58 students in experimental group A and 56 students in experimental group B. Experimental group A received a blended version of RISE comprising synchronous and asynchronous methods, take-home tasks, and quizzes (Table 1). Each week, participants had access to educational materials in the form of videos and reading materials. A program guide including the schedule, helplines, and take-home tasks ranging from reflective practices to practical exercises and quizzes relating to that week’s topic was provided to reinforce students’ learning. Finally, three virtual face-to-face discussions were conducted over Zoom, and a forum was made available to facilitate discussions. Experimental Group B was an asynchronous group that only had access to the videos. The contents of the materials were the same in both groups.

### 2.4. Procedure

Following ethics approval, participants from both experimental groups were purposively sampled using the maximum variation technique [51] based on demographic (e.g., age, gender, and ethnicity), academic (e.g., faculty and seniority), participation (e.g., completion rate), and resilience scores. Students of various completion rates were recruited to gather richer insights into the facilitators and barriers to participating in the RISE program. The overall mean score for the resilience outcome using the 25-item Connor Davidson Resilience Scale [52] was 72.27 (range: 46 to 97) at the post-intervention time point. Hence, participants with higher or lower scores were recruited to appreciate how the RISE program influenced their resilience. Following the completion of RISE, selected participants were invited through email to join an individual online face-to-face semi-structured interview via Zoom.

Three authors (DA, LY, and SS) developed the semi-structured interview guide concerning the process evaluation framework [38]. Based on the process evaluation framework [38], two domains relating to the (1) overall experience (e.g., experiences of the RISE program), and (2) contents of the intervention (e.g., understanding the effects of the resilience strategies) were developed. The initial guide was circulated to all members of the research team for review. Two pilot interviews were conducted to ensure that the interview guide is comprehensible. The eventual interview guide was shortened and adjusted for clarity (Table 2).

The audio- and video-recorded interviews were conducted at a time convenient to the participants via Zoom. The interviewer (NO) was a male honor student trained in qualitative research methods and had experience in conducting qualitative interviews. To reduce the possibility of social desirability and response bias, the interviewer was not involved in the delivery of the RISE program. Debriefing sessions were held with the first author (DA) to ensure that the interviewer remained consistent during the data collection.

A total of 20 students were recruited in this study. There were three dropouts due to the inability to commit to the interviews resulting in a total of 17 participants. All participants were reimbursed with SGD 10 for their time. The mean duration of the interviews was 37.47 min (range: 26 to 57 min). Data analysis occurred simultaneously during the data collection to assess for data saturation. Data saturation, where no new information emerged from the interviews was achieved at the 15th participant [53]. Two additional interviews were conducted to confirm saturation, hence a total of 17 students participated in this study.

### 2.5. Data Analysis

The audio- and video-recorded interviews were transcribed verbatim by two researchers (JZ and NO) using Microsoft Word. The transcripts were subsequently checked by one author (DA) for verbatim accuracy. All identifiable information was removed, and pseudonyms were used to replace any mentioned names to ensure anonymity. Participants were provided with a copy of the transcript and were invited to provide clarifications.

Thematic analysis [54], which was a six-stage process, was conducted using an inductive approach by three authors (DA, NO, and JC). First, the authors familiarized themselves with the data by reviewing the transcripts and interview videos simultaneously. Second, all authors individually developed three sets of codebooks. Codes were derived inductively from participants’ narratives using an open and iterative semantic coding process. Third, all three sets of codebooks were brought together for comparison, and a fourth author (LY) was involved in the discussion.

**Figure 2:**
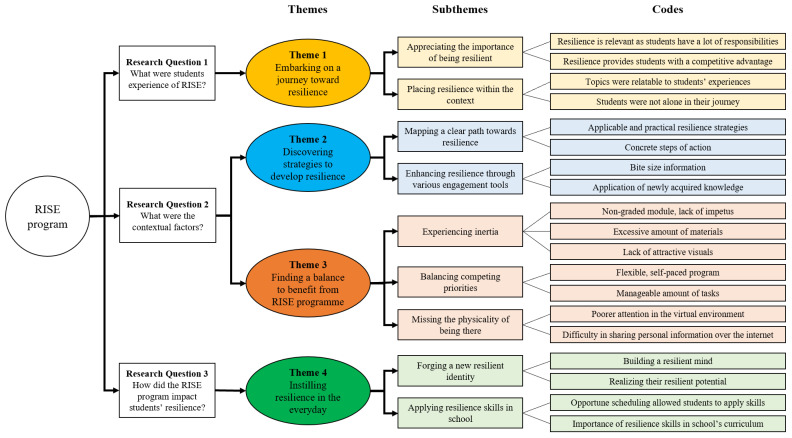
Coding tree.

A total of 183 codes were identified from the transcripts, and these codes were condensed and compiled using Microsoft Excel. Fourth, the codes were further analyzed using the constant comparative method [55] by reviewing the similarities and differences regarding the transcripts to ensure an accurate interpretation. Fifth, following consensus on the developed codes, the data were reduced by removing overlapped and redundant codes. Sixth, a total of nine subthemes and four themes were developed. The coding tree is depicted in Figure 2. All members of the team reviewed the themes. Participants were invited to review the themes to ensure the themes were representative of their narratives.

### 2.6. Rigor

Several strategies were used to establish and ensure trustworthiness based on the criteria of credibility, transferability, dependability, and confirmability [56]. Credibility was established through the piloting of the interview guide, member checking, and prolonged engagements with the participants [56]. A dense description of the sample and setting allowed researchers to gain a deeper insight into the situational uniqueness, thus ensuring transferability [56]. In addition, the participation of three independent researchers during the data analysis was done to determine dependability. Finally, the interviewer kept the recorded interviews and reflexivity journals to maintain confirmability.

### 2.7. Ethical Considerations

Ethical approval was obtained from the university’s institutional review board (NUS-IRB-2021-594) before the study commenced. Students were provided with explanations about voluntary participation, confidentiality, the right to withdraw, and potential risks and benefits. Students were further reassured that declining participation would not lead to any penalties or any differences in their academic grades. Consequently, written informed consent was obtained from each student. 

## 3. Results

Seventeen students participated in this study. The mean age was 22.53 (4.39) with a range of 19 to 38 years. The majority of the participants were ethnic Chinese (88.23%) and female (58.82%). Participants were from a variety of faculties and seniorities. The mean resilience score of the participants in this study was 70.59 (15.10). Nine participants were from experimental group A; eight, group B. The average completion rate of the RISE program was 85%. The total possible number of minutes of engagement for groups A and B was 431 min and 251 min, respectively. The details of the participants’ characteristics are in Table 3.

**Figure 3:**
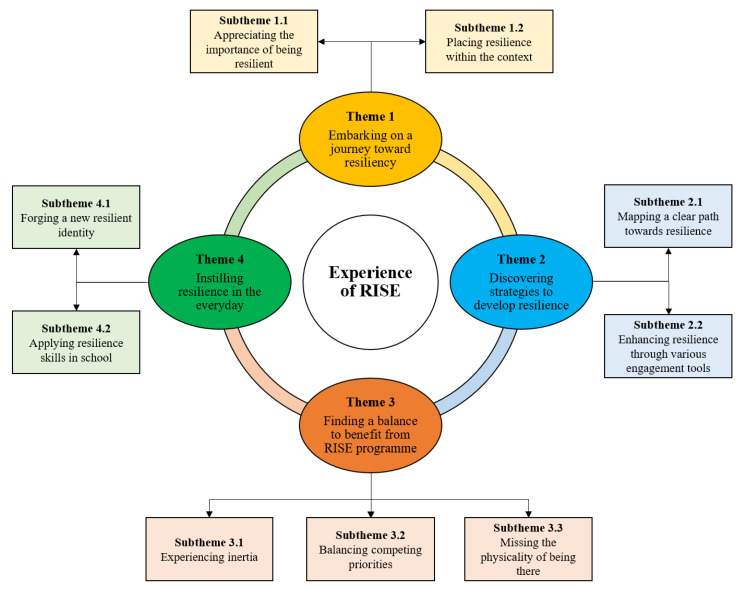
Students’ experience of the RISE program.

The thematic analyses based on participants’ narratives unveiled how the RISE program supported their resilience journey (Figure 3). They additionally described how certain features of the RISE program facilitated or limited their ability to become resilient. Their descriptions are elaborated in four themes: (1) embarking on a journey toward resiliency, (2) discovering strategies to develop resilience, (3) finding a balance to benefit from RISE, and (4) instilling resilience in the everyday.

### 3.1. Embarking on a Journey toward Resiliency

The first theme provides an insight into students’ experiences of the RISE program. Students likened their experience in the RISE program to the start of their journey toward resiliency. The RISE program provided students with resilience-enhancing skills within a familiar context. Their journey to resilience was elaborated in two subthemes: (1) appreciating the importance of being resilient and (2) placing resilience within the context.

#### 3.1.1. Appreciating the Importance of Being Resilient

Students described their life in university as one filled with multiple challenges and conflicting priorities. In light of those experiences, they appreciated the RISE program as it highlighted the importance of being resilient as a tool to develop resistance against adversities. In a third-year medical student’s narrative:

“*To build up that [resilience] skill because I feel it is more relevant now as an upper university year student where you have to deal with a lot more responsibilities, knowledge, and pressure*” (P3, Female, Medicine, Year 3, Group A)

Recognizing the importance of being resilient, students embarked on a journey in search of opportunities to enhance their resilience.

“*The reason I signed up for the [RISE] program was because this semester was going to be very hectic for me, and I was thinking about how to be more resilient and stronger … that’s something I wanted to adopt*” (P12, Female, Science, Year 3, Group B)

Further, through the RISE program, students appreciated the importance of being resilient for other reasons. Specifically, being resilient was viewed as a competitive advantage, a trait deemed to be valued by others:

“*I acknowledge the value because I think that it [referring to being resilient] creates a competitive advantage for me if I am more resilient. Especially if every one of us faces the same problem and I can get out of it much faster, actually it proves that I have more capabilities*” (P7, Male, Business, Year 1, Group A)

#### 3.1.2. Placing Resilience within the Context

Students appreciated how the RISE program is situated in contextually relevant content and how it resonates with their actual experiences in school. In particular, students credited the mirroring effect through the videos and Zoom seminars and gained perspectives when they related to how the examples are contextualized. A student shared:

“*The topics that were brought up in this program were very relatable to me. In most videos, they brought up the different challenges that students could face in university and how we could resolve these challenges*” (P4, Female, Nursing, Year 3, Group A)

The virtual face-to-face seminars also provided students with a platform to normalize their experienced stresses and realize that these experiences were not unique to themselves. One computing student mentioned:

“*The discussions [referring to the zoom seminars], which were being facilitated, as I got to learn a lot from other people’s experiences. It made me realize that I wasn’t the only one having these issues*” (P9, Male, Computing, Year 3, Group A)

Apart from the use of contextually relevant scenarios, participants valued how the resilience-enhancing strategies were correspondingly applicable to their experienced challenges. In the interview of an arts student:

“*I remember when the facilitator talked about this tip. I was just, like, that is very true. That’s another thing I quite appreciate because he was very specific in saying that when you share, don’t just share it with anybody, you must be someone who can empathize with the situation, which is something that makes a lot of sense*” (P17, Female, Arts, Year 5, Group B)

The aforementioned perceived benefits were not the same for all though as some students contended with their difficulties to relate the proposed strategies. One student shared:

“*The content about coping and creating positivity … I find it slightly preaching in some ways, like ‘this is the information’ … If it writes that you must be positive, how do you say no to that?*” (P14, Male, Architecture, Year 4, Group B)

### 3.2. Discovering Strategies to Develop Resilience

As participants navigated through the RISE program, they started to discover how these strategies came together to build their resilience. They drew inspiration from the variety of learning engagement tools in aiding their development of resilience. The mechanisms in which the RISE program influenced students’ resilience were illustrated in two subthemes: (1) mapping a clear path toward resilience and (2) enhancing resilience through various engagement tools.

#### 3.2.1. Mapping a Clear Path toward Resilience

In students’ journey of becoming resilient, they cherished how the RISE program provided concrete examples of enhancing their resilience, which was distinct from other available training. The instructional steps eliminated their need to seek professional help as they were able to map their path toward resilience. In one student’s narrative:

“*The facilitator gave great points like giving examples on how to cope or giving concrete examples on how to work through your negative thoughts. That is very helpful and very hard to find on the internet and in just any lecture … Because if you go and talk to a therapist, but then you just talk. However, you want help, like you want to know what are the concrete step that you can take, what can you do*” (P10, Female, Nursing, Year 3, Group B)

Students additionally appreciated how these strategies were practical and could be easily and readily applied in their daily lives. One science student recounted:

“*I think the tips that the speaker shared were also really good because they weren’t like impractical … they weren’t too out of reach. It was still something that I could incorporate into my daily life*” (P8, Female, Science, Year 3, Group A)

For the abovementioned reasons, participants were able to utilize these strategies and thus enhance their resilience. In one’s student interview:

“*I start to have a positive portfolio. So, I had a list that time. I had a list like this. I wrote down on this thing this specific thing, who helped me on this, like who helped me bought something, I wrote it all down*” (P7, Male, Business, Year 1, Group A)

#### 3.2.2. Enhancing Resilience through Various Engagement Tools

Considering the various learning styles, the RISE program provided multiple avenues to support different types of learners. Students enhanced their resilience through the availability of different types of engagement tools. Participants in both groups alluded to the bite-size information in the form of short videos as a manner in which they learned how to be resilient. A science student shared:

“*I liked how the videos were broken down into small parts … it was easy to have bite-sized information each time*” (P13, Male, Science, Year 3, Group B)

The majority of the students in experimental group A also identified how the provided quizzes were useful in supporting their learning. Specifically, the provision of feedback based on their selected responses was appreciated. One student shared:

“*I felt that the quizzes were helpful as it even provided feedback on the responses, which was helpful for the participants*” (P9, Male, Computing, Year 3, Group A)

Among students with access to various self-assessment tools and weekly tasks, they viewed them as an opportunity to apply their newly acquired skills to practice. This chance was particularly useful as students may not be experiencing any form of specific challenges and were unable to use it in an actual situation. One student shared:

“*The take-home assignment is good because it let you apply the skills you’ve learned in that specific week, so it kind of gets you to categorize, like ‘okay, this week I learn about this topic, so how am I going to apply this to my life?’ It teaches/guides me in the application process, rather than I go and draw the links myself*” (P2, Female, Nursing, Year 2, Group A)

### 3.3. Finding a Balance to Benefit from RISE

The RISE program started during the university’s vacation period (winter break) and concluded in the third week of the new academic term. As students began a new semester, they contended with academic work while attempting to complete the RISE program. Coupled with other numerous competing priorities, students verbalized several difficulties in keeping up. Three subthemes emerged: (1) experiencing inertia, (2) balancing competing priorities, and (3) missing the physicality of being there. All these described the inhibitors for participating in the RISE program.

#### 3.3.1. Experiencing Inertia

Although the majority of the students were motivated to participate in the RISE program, it was not a credit-bearing module, and this might have been a disincentivizing factor. This has led to some students experiencing a form of inertia:

“*I think it was more like personal, like laziness because there wasn’t like a consequence … so I think there wasn’t an impetus to make me finish the stuff*” (P3, Female, Medicine, Year 4, Group A)

Inertia was also evidently observed when few students in the RISE program tapped into the reading resources such as lecture notes and supplementary readings. Students cited that the readings were “extra” and “unnecessary” because they got most of the resilience skills from the videos. Their lack of motivation to review additional reading materials was not unique to the RISE program:

“*I didn’t read much for the additional readings … because if I read, it’s gonna take a lot of time for me. I feel like ‘oh my God,’ it’s so much. I do not even read my additional reading for my module*” (P7, Male, Business, Year 1, Group A)

To reduce the inertia and spark their reading interests, students highlighted their preference for more attractive visuals that were different from those encountered in their academic work. One student mentioned:

“*What entices me would be something with colors and diagrams, not academic diagrams but those that are more visually pleasing to a non-academic audience*” (P11, Male, Arts, Year 4, Group B)

#### 3.3.2. Balancing Competing Priorities

The majority of students mentioned how they had to juggle academic work and remain engaged with the RISE program. Nevertheless, students attributed several design features that enabled them to balance their competing priorities. The familiarity with the RISE platform was an enabler for students, without the need to enter an alternative platform or recall log-in details:

“*It is a good thing because I am familiar with LumiNUS. It is easy to access and use the features. There is no need to save a tab and log in with another ID and password on a different platform*” (P8, Female, Science, Year 3, Group A)

In addition, participants valued the flexible nature of the RISE program, allowing them to review the materials at their own pace. This helpful feature allowed them to keep up. In one’s student interview:

“*I like how the videos can be reviewed at my own pace because I kind of have like a fixed timetable schedule, so if there was a fixed Zoom that I have to go through, then I’m quite afraid that I would not be able to turn up*” (P15, Female, Year 1, Science, Group B)

Finally, students also found that the workload was manageable and appreciated how the RISE program was planned with consideration of the academic timetable.

“*I think they [referring to the tasks] were very manageable because the load was heavier before the semester started. Then as we go through the semester, it was just like you only need to do the quiz and upload. So, I would say the customization reflected an understanding of our workload*” (P1, Male, Engineering, Year 4, Group A)

#### 3.3.3. Missing the Physicality of Being There

Owing to COVID-19 and the social isolation measures, the RISE program was designed as a remote learning training with all forms of synchronous and asynchronous communication taking place over the virtual platform. Although synchronous seminars via Zoom were available to students in experimental group A, students preferred the physical presence. More prominently, students in experimental group B cited that interacting over a digital platform has reduced their ability to focus. In one student:

“*The physicality of being there. Because right now if I am in front of my computer, I might pause the video to go somewhere else. Although I spend the same amount of hours there, it takes some time for my mind to get back. Whereas for a physical space, we are kind of forced to be in it, and we can’t go elsewhere*” (P14, Male, Architecture, Year 4, Group B)

Some students highlighted how being physically present allowed them to observe non-verbal cues that are not available through virtual means. The physical presence was central to their levels of engagement:

“*[Physical] Face-to-face sessions allow us to see the other person’s face, body movements, posture... It is more dynamic and engaging compared with virtual zooms*” (P11, Male, Arts, Year 4, Group B)

In addition, discussion sessions via Zoom comprised topics that detailed participants’ journey to resilience, which often included personal anecdotes or experiences. Sharing these details over a digital platform was a challenge for some students, so a physical session was preferred. In one’s student interview:

“*I just felt very awkward on Zoom.... Sharing about our resilience journey might be quite personal... I’m not sure if everyone could share such personal stuff with people we are meeting for the first time on Zoom … having discussions in person might help facilitate that*” (P5, Female, Arts, Year 2, Group A).

### 3.4. Instilling Resilience in the Everyday

As students continued in the RISE program, they began to understand how the respective resilience-enhancing strategies gel together. Through their narratives, students were able to apply these resilience skills in their everyday lives, ranging from personal to school. Two subthemes illustrated their application of resilience skills: (1) forging a new resilient identity and (2) applying resilience skills in school.

#### 3.4.1. Forging a New Resilient Identity

This subtheme depicted how students with their newly equipped resilience went on to make sense of how these skills fit in their lives. Different skills ranging from emotional (e.g., creating positivity) to practical coping tips (e.g., time management) were imparted, so students were able to use the skill they needed to make positive changes in their lives. In one student’s narrative:

“*After attending this RISE program, I learned to look at the bigger picture or the more intangible side about my mental resilience... This kind of change in mindset is something that goes hand in hand with resilience or having a resilient mindset*” (P14, Male, Architecture, Year 4, Group B)

Other students appreciated equipping themselves by keeping a set of “tools” that they might refer to in times of adversities. In doing so, they came up with a new resilient identity.

“*Knowing how to better manage emotions and time, what can we do, that just gets us through challenging times but [to] help us be more, you know, physically and mentally stronger as well as fitter. I think that’s a lot healthier than sometimes when we just focus on getting through tough times. Instead, we are getting ready for next time*” (P13, Male, Science, Year 3, Group B)

Following the RISE program, several students realized that they were resilient before participating in the program. This group of students found a resemblance between the imparted resilience-enhancing strategies with their existing behaviors. Notwithstanding, the RISE program acted as a reinforcement of their resilient identity:

“*After I joined the program, I realized ‘oh, I am pretty decently resilient’ … Now I’m more confident. I know I am resilient, and I own it. I feel more courageous when I’m faced with a challenge*” (P10, Female, Nursing, Year 3, Group B)

#### 3.4.2. Applying Resilience Skills in School

Given that a considerable portion of the RISE program focused on building students’ resilience within an academic setting, students were able to apply resilience skills in school. Students also attributed to the opportune scheduling of the RISE program, allowing them to transfer the skills to an actual situation. In two students’ narratives:

“*This resilience program started around mid- to almost end of December, so it was half of the winter [break] … after that when studies start to come in that’s when the actual application of resilience came, like how to control or juggle a few things*” (P7, Male, Business, Year 1, Group A)

“*Before the program, I never really journal.... But after the facilitator introduced it, I started doing it more often. It kind of helps us buffer the stress.... [It taught me to] be happy because I have something to be grateful for even though FYP [Final Year Project] was very difficult*” (P8, Female, Science, Year 3, Group A)

Along with their new resilient identities, students started applying and instilling these resilience skills in their everyday lives. For one student, these newly acquired skills were also useful to the people around:

“*The worst-case scenario, best-case scenario module was applicable not just for me in my personal life but also in helping others and trying to get them through whatever it is that they are going through. So, the RISE program did not just reach me but also the people around me. It was really helpful for me*” (P10, Female, Nursing, Year 3, Group B)

Through their experiences in the RISE program and recognizing its importance, students expressed the need for the RISE program to be formally included as part of the university’s curriculum. As shared by one student:

“*I think this [referring to resilience skills] is something we should just be taught. Like, we shouldn’t need to go through a separate program to do this*” (P13, Male, Science, Year 3, Group B).

## 4. Discussion

This study adopted a qualitative process evaluation approach to explore undergraduate students’ experiences of the RISE program. To the authors’ knowledge, information about resilience training using process evaluation methods is limited, specifically within an Asian context. The thematic analysis unveiled four themes that highlighted how the implementation of RISE, its casual mechanisms, and contextual factors influenced students’ resilience. Students generally appreciated the use of contextually relevant content and the availability of various learning engagement tools. Students also credited the RISE program for the provision of clear and practical techniques that allow them to enhance their resilience. However, some students experienced inertia and competing priorities that limited their participation. Given that the intervention was delivered solely via a digital platform, students verbalized poorer engagement with the RISE program.

The positive effects of digital resilience training in enhancing students’ resilience found in this study were similar to those reported in other studies [57,58]. Participants attributed this outcome to several reasons. First, participants appreciated how the training was situated within their context and that they were able to relate to the content. This reason could be explained through an experiential learning theory [59,60]. As the RISE program included interviews with other students, participants were able to create knowledge with the transformation of experiences through two mechanisms, reflective observation and active experimentation [60]. Through the videos, participants were able to relate to the experiences of the speakers who were current students or recent graduates. Through the take-home tasks and the start of the academic semester in the third week of the RISE program, students were able to actively experiment with their newly acquired skills. Regarding the characteristics of the current generation of undergraduate students, studies have highlighted that these students prefer such experiential learning methods and do not value traditional lecture styles [61,62]. These findings imply that didactic approaches should not comprise solely traditional forms of lectures but also include a variety of mediums (e.g., conversations or podcasts) to impart knowledge.

Second, the RISE program comprised virtual discussions and forums, and these were valued aspects of the training. More importantly, these features, as a form of the dialogic process, provided students with an opportunity to learn from others, which were found to be essential for resilience training [21]. Central to the social learning theory [63], one’s social environment can influence their learning. In addition, students may learn through vicarious experiences by observing people around them [63]. Through the virtual discussions, students were grouped with students from different faculties and seniorities, enabling them to learn resilience skills by modeling after their peers [64]. Sharing over an online forum allows students to reflect better [65]. This important element is useful for building resilience as engaging reflective practices can better enhance students’ resilience [50]. Given the importance of social learning within the context of resilience training, incorporating elements that facilitate social engagements is crucial.

Third, students appreciated the use of various engagement tools such as videos, quizzes, and practical exercises in the RISE program. The pre-recorded videos were the primary mode of sharing resilience knowledge using didactic modes and were made available online for students to view at their own pace and customize their own learning. This approach allows students to gain autonomy and control of the schedule which was found to maximize their learning [66,67]. Quizzes are commonly used in formative assessments to assess students’ learning and knowledge [68]. Unlike quizzes typically designed as one-sided that require participants’ responses, the unique feature of RISE’s quizzes was it being interactive with the inclusion of feedback. The feedback allowed students to understand why the chosen response was correct or wrong. This feature is important as it enhances the acquisition and retention of knowledge [69]. The RISE program also provided students with practical exercises such as reflective practices and worksheets. Practical exercises are often found in different types of psychosocial interventions such as mindfulness or behavioral therapies [70,71]. Given that being resilient requires students to adopt or make changes in their existing behaviors or thought processes, practical exercises provide an avenue for students to apply their newly acquired skills. More importantly, with further application and practice, students can achieve better long-term outcomes [72].

However, despite the numerous merits associated with the RISE program, students experienced several barriers over the course of the six weeks of training. Students described several drawbacks of RISE, including the presence of inertia, competing priorities, and the digital platform. The effectiveness of the RISE program could have been compromised by the inertia experienced by students as well as the lack of physical interaction. Experiencing inertia was similarly reported by students using digital learning platforms [73,74]. Students’ inertias were also exacerbated for several reasons, such as having an excessive number of readings, unattractive diagrams, and it being not a graded module. With these factors in mind, future training programs need to balance the number of reading materials for a blended learning module and complement it with attractive visuals. Although grade-free systems encourage independent learning, [75] this was not the same for participants in this study. This could be due to the lack of incentives or motivation when no grades were involved [76].

The RISE program started during the university’s vacation break and ended on the third week of the new academic semester. Hence, that students verbalized competing priorities and placed more emphasis on their graded academic work was expected. This was also observed based on the declining completion rate when the semester began. This finding was similarly reported by Rasheed [77]. Nonetheless, students were reminded to complete the RISE program as it was on the same learning platform as their academic modules. This provides an insight for future student wellness programs to embed these programs into a system that students frequently use or are familiar with so as to improve their learning and interaction [78,79]. Given the widespread use of smartphones, learning platforms can be made available on smartphones to foster students’ engagement [66].

The use of digital platforms is often required to enhance the scalability and accessibility of psychological interventions [80,81]. However, disengagement is also common with interventions hosted over a digital platform [81], especially in less expensive formats such as lectures [82]. This was similarly verbalized in this study, particularly among students in experimental group B (asynchronous version) where the lack of physical engagement affected their engagement with the RISE program. Although interactivity is a concern, the findings from this process evaluation suggested that it has to be balanced appropriately to ensure that it does not compete with students’ academic work. For instance, digital training embedded in gamification concepts may be a plausible strategy to increase engagement [83,84] and is preferred by the current generation of students [85]. However, more work is required to confirm that the investments (e.g., costs and time) in game production can yield a superior learning outcome.

The RISE program did not bear any modular credits or contributed to students’ academic grades, and this led to sentiments of disincentivization among the students. Such a sentiment was also seen in massive open online courses, where participant dropout remains high largely due to the lack of incentives [86,87]. As resilience training gains recognition as a form of self-help program to enhance students’ well-being, students’ feelings of being disincentivized should be considered. Educational institutes may consider mandating the course as part of the university curriculum, for instance, as part of a freshmen orientation program or offering modular credits as an elective course. Such a consideration is significant given that assessments have been proposed to drive learning among voluntary courses [88,89].

Given that participants had to reflect and share personal information about their vulnerabilities in the course of their journey toward resilience, they also verbalized difficulties in sharing intimate information through an online platform. This finding was surprising as individuals are more willing to share intimate information over online platforms as opposed to a face-to-face discussion [90]. Conversely, emotional drivers, for instance, shame or fear, may reduce students’ willingness to share over an online platform [91]. These findings provided several key considerations for future trials. First, should resilience training be formalized into the university curriculum, and should the frequency of discussions be increased to foster familiarity and rapport among students? Second, alternative platforms such as online forums with an anonymous function may be used to encourage the sharing of more sensitive information. Finally, grouping students from the same faculty who are already familiar with one another may ease them into engaging with sensitive topics.

### 4.1. Implications for Practice and Research

Based on the findings of this process evaluation, several key considerations for a future training program are put forward. First, designing the interactivity of the RISE program warrants greater emphasis. Although resource intensive, students value synchronous communication through “live” discussion sessions with the purpose of greater engagement and social learning. With competing priorities, alternative modes of synchronous communication should be identified. Examples are discussions through forums or increasing engagement through videos by embedding gamification or interactive components such as quizzes. More work is needed to examine how interventions can engage and stimulate students in a digital platform while ensuring that the mental well-being training remains accessible and acceptable to students.

Second, although students saw the value in being resilient, they were inundated by the vast number of readings and tasks in the RISE program. Educators need to review the number of materials that students are required to review. As the RISE program was not credit-bearing and students experienced inertia in completing the tasks, reviewing how mental wellness programs may be integrated into the university curriculum is worthwhile. For instance, with the ultimate goal of providing students with protected time, offering mental wellness programs as electives with credits can enable students to fully immerse themselves in the program. Alternatively, future training programs may consider making quizzes and practical tasks optional to reduce the burden on students.

Third, students attributed their increase in resiliency due to a variety of skills (e.g., positivity and mindset). Future studies should draw an in-depth understanding of the interactions between these skills. Specifically, it will be useful to know if these skills work in isolation or in synergy. This will have practical implications for the development of future resilience programs. Finally, considering that this study comprised two experimental groups, future trials should consider the inclusion of a usual care comparator. This will be helpful in confirming the effects of the RISE program on students’ resilience. Further, it will also be important to conduct larger-scale RCTs among various institutions of higher learning, this will be critical in ensuring that the RISE program is generalizable among a wider population.

### 4.2. Limitations

The findings of this study have several limitations. First, this study is limited to one university in Singapore. Second, considering that only 17 participants (n = 14.9%) from the RISE program participated in this qualitative study, the findings may not be representative of all participants. Nevertheless, the purposive sampling approach ensures that findings are representative of a diverse student population based on ethnicities, faculties, and seniorities. Third, students’ insights are limited to one resilience training program, so the findings may not be transferable to other resilience programs. Nonetheless, students’ recommendations and suggestions may provide pedagogical insights for future resilience programs using either the synchronous or asynchronous method.

## 5. Conclusions

Digital platforms are increasingly accessible and leveraging a digital platform to increase the scalability of a training program remains promising. With the increasing popularity of using digital platforms, for instance, blended learning in universities, the urgent question of “what and how much to blend” remains. The findings of this process evaluation expand the existing literature by providing additional information about participants’ experiences of an asynchronous and synchronous version of a resilience training program. The study also highlights the mechanisms of the RISE program in enhancing students’ resilience.

## Figures and Tables

**Table 1:** Resilience Skill Enhancement (RISE) Program.

Week	Session	Format	Topics	Discussion	Take-Home Tasks	Overall Duration
1	Introduction to resilience and embracing change	Lectures	1. Introduction to resilience (9 min) 2. Embracing change (9 min)	Zoom seminar (60 min)	1. Quiz 2. Reflective practice – Identifying sources of resilience	92 min
		Interview with current students	3. Narratives from a resilient individual (14 min)			
2	Coping strategies	Lectures	1. Coping strategies (11 min) 2. Resilient coping (6 min) 3. School-based services (11 min)	Forum	1. Quiz 2. Practical exercise – Applying and evaluating coping strategies	50 min
		Interview with current students	4. Coping with academics in NUS (22 min)			
3	Creating positivity	Lectures	1. What is positivity (2 min) 2. Benefits of positivity (2 min) 3. Strategies to increase positivity (18 min)	Forum	1. Quiz 2. Practical exercise – Finding positivity in the everyday life	36 min
		Interview with current students	4. Experiences of creating a positive mindset (14 min)			
4	Shifting mindsets	Lectures	1. Mindsets (2 min) 2. Fixed and growth mindsets (10 min) 3. Activating a change in mindsets (11 min)	Forum	1. Quiz 2. Practical exercise – Applying a growth mindset in conservations	23 min
5	Building social competency	Lectures	1. Basic communication skills (7 min) 2. Managing difficult relationships (6 min) 3. Building meaningful relationships (9 min) 4. Conflict resolution (6 min)	Zoom seminar (60 min)	1. Quiz 2. Self-assessment survey – Identifying your love, anger, and apology languages	88 min
6	Preparing for the future	Interview with content experts	1. Financial management (25 min) 2. Preparing for job search and interviews (24 min) 3. Becoming an entrepreneur (16 min)	Zoom seminar (60 min)	1. Quiz 2. Self-assessment survey – Career preference quiz	142 min
		Interview with current students	4. Financial planning for undergraduates (17 min)			

Note: Total duration for all videos is 251 min.

**Table 2:** Semi-structured interview guide.

Domains	Questions
Overall experience	1. May you describe your experience of the RISE training? 2. Did you experience any issues with the training platform? 3. How did you feel about the duration of the training program? 4. How was your experience with regards to the specific weekly sessions? 5. How may we improve the training program?
Contents of the intervention	1. Was there any particular session that you found useful? 2. Was there any particular session that was not useful? 3. Were there any resilience enhancing strategies that we did not cover in this training? 4. Were there any factors that affected your participation in the RISE training?

**Table 3:** Participant characteristics.

ID	Socio-Demographic Characteristics							RISE Program Characteristics			
	Gender	Age	Ethnicity	Course	Seniority *	Resilience Score	Interview Duration	Group	Total Time ^#^	Submission of Take-Home Tasks	Overall Completion Rate
P1	Male	24	Chinese	Engineering	4	52	40	A	208 min	100%	71.43
P2	Female	20	Chinese	Nursing	2	77	41	A	429 min	100%	100
P3	Female	22	Chinese	Medicine	4	68	26	A	80 min	16.7%	38.1
P4	Female	22	Burmese	Nursing	3	88	37	A	349 min	100%	71.43
P5	Female	20	Chinese	Arts	2	93	38	A	405 min	100%	95.24
P6	Female	20	Chinese	Computing	2	68	34	A	162 min	100%	76.19
P7	Male	19	Chinese	Business	1	90	57	A	374 min	100%	85.71
P8	Female	22	Chinese	Science	3	86	30	A	433 min	100%	100
P9	Male	38	Chinese	Computing	3	60	30	A	434 min	100%	100
P10	Female	22	Chinese	Nursing	3	81	32	B	251 min	NA	100
P11	Male	24	Chinese	Arts	4	68	35	B	196 min	NA	85
P12	Female	21	Indian	Science	3	73	38	B	186 min	NA	83.3
P13	Male	24	Chinese	Science	3	54	45	B	225 min	NA	92
P14	Male	24	Chinese	Architecture	4	81	36	B	219 min	NA	90
P15	Female	19	Chinese	Science	1	65	33	B	205 min	NA	88
P16	Male	19	Chinese	Science	2	39	45	B	187 min	NA	83.5
P17	Female	23	Chinese	Arts	5	57	40	B	198 min	NA	85.5

* Seniority refers to students’ year of study at the university at the point of participating in the RISE program. ^#^ Total time is the tabulation of the total number of minutes for videos (251 min) and zoom seminars (180 min).

## Data Availability

The data presented in this study are available on request from the corresponding author. The data are not publicly available due to ethical reasons.

## References

[1] Dewart G., Corcoran L., Thirsk L., Petrovic K. (2020). Nursing education in a pandemic: Academic challenges in response to COVID-19. Nurse Educ. Today.

[2] Aruguete M.S. (2017). Recognizing challenges and predicting success in first-generation university students. J. STEM Educ. Innov. Res..

[3] Reddy K.J., Menon K.R., Thattil A. (2018). Academic stress and its sources among university students. Biomed. Pharmacol. J..

[4] Yang C., Chen A., Chen Y. (2021). College students’ stress and health in the COVID-19 pandemic: The role of academic workload, separation from school, and fears of contagion. PLoS ONE.

[5] Kaisara G., Bwalya K.J. (2021). Investigating the E-learning challenges faced by students during COVID-19 in Namibia. Int. J. High. Educ..

[6] Maatuk A.M., Elberkawi E.K., Aljawarneh S., Rashaideh H., Alharbi H. (2021). The COVID-19 pandemic and E-learning: Challenges and opportunities from the perspective of students and instructors. J. Comput. High. Educ..

[7] Adedoyin O.B., Soykan E. (2020). COVID-19 pandemic and online learning: The challenges and opportunities. Interact. Learn. Environ..

[8] Lemay D.J., Bazelais P., Doleck T. (2021). Transition to online learning during the COVID-19 pandemic. Comput. Hum. Behav. Rep..

[9] Barrot J.S., Llenares I.I., Del Rosario L.S. (2021). Students’ online learning challenges during the pandemic and how they cope with them: The case of the Philippines. Educ. Inf. Technol..

[10] McCloud T., Bann D. (2019). Financial stress and mental health among higher education students in the UK up to 2018: Rapid review of evidence. J. Epidemiol. Community Health.

[11] Prowse R., Sherratt F., Abizaid A., Gabrys R.L., Hellemans K.G.C., Patterson Z.R., McQuaid R.J. (2021). Coping with the COVID-19 pandemic: Examining gender differences in stress and mental health among university students. Front. Psychiatry.

[12] Lasheras I., Garcia-Garcia P., Lipnicki D.M., Bueno-Notivol J., Lopez-Anton R., Camara C., Lobo A., Santabarbara J. (2020). Prevalence of anxiety in medical students during the COVID-19 pandemic: A rapid systematic review with meta-analysis. Int. J. Environ. Res. Public Health.

[13] Quek T.T., Tam W.W., Tran B.X., Zhang M., Zhang Z., Ho C.S., Ho R.C.H. (2019). The global prevalence of anxiety among medical students: A meta-analysis. Int. J. Environ. Res. Public Health.

[14] Tung Y.-J., Lo K.K.H., Ho R.C.M., Tam W.W. (2018). Prevalence of depression among nursing students: A systematic review and meta-analysis. Nurse Educ. Today.

[15] Puthran R., Zhang M.W.B., Tam W.W., Ho R.C.M. (2016). Prevalence of depression amongst medical students: A meta-analysis. Med. Educ..

[16] Liu C.H. (2019). The prevalence and predictors of mental health diagnoses and suicide among US college students: Implications for addressing disparities in service use. Depress. Anxiety.

[17] Dekker I., De Jong E.M., Schnippers M.C., Brujin-Smolders M., Alexiou A., Giesbers B. (2020). Optimizing students’ mental health and academic performance: AI-enhanced life crafting. Front. Psychol..

[18] Rose T., Lindsey M.A., Xiao Y., Finigan-Carr N.M., Joe S. (2017). Mental health and educational experiences among black youth: A latent class analysis. J. Youth Adolesc..

[19] Muller A.E., Hastad E.V., Himmels J.P.W., Smedslund G., Flottorp S., Stensland S.O., Stroobants S., Van de Velde S., Vist G.E. (2020). The mental health impact of the COVID-19 pandemic on healthcare workers, and interventions to help them: A rapid systematic review. Psychiatry Res..

[20] Go D.-S., Kim Y., Paik J., Roh S., Yoon S. (2020). A comparison of disease burden and the government budget for mental health in Korea. J. Ment. Health.

[21] Ang W.H.D., Lau S.T., Cheng L.J., Chew H.S.J., Tan J.H., Shorey S., Lau Y. (2022). Effectiveness of resilience interventions for higher education students: A meta-analysis and metaregression. J. Educ. Psychol..

[22] Brewer M.L., Kessel G., Sanderson B., Naumann F., Lane M., Reubenson A., Carter A. (2019). Resilience in higher education students: A scoping review. High. Educ. Res. Dev..

[23] Ang W.H.D., Shorey S., Hoo M.X.Y., Chew H.S.J., Lau Y. (2021). The role of resilience in higher education: A meta-ethnographic analysis of students’ experiences. J. Prof. Nurs..

[24] Pooley J.A., Cohen L. (2010). Resilience: A definition in context. Aust. Community Psychol..

[25] Ong A.D., Bergeman C., Chow S.-M. (2010). Positive emotions as a basic building block of resilience in adulthood. Handbook of Adult Resilience.

[26] Leys C., Arnal C., Wollast R., Rolin H., Kotsou I., Fossion P. (2020). Perspectives on resilience: Personality trait or skill?. Eur. J. Trauma Dissociation.

[27] Masten A.S. (2015). Ordinary Magic: Resilience in Development.

[28] Mancini A.D., Bonanno G.A. (2009). Predictors and parameters of resilience to loss: Toward an individual differences model. J. Personal..

[29] Van Breda A.D. (2001). Resilience Theory: A Literature Review.

[30] Van Breda A.D. (2018). A critical review of resilience theory and its relevance for social work. Soc. Work..

[31] Rutter M. (2007). Resilience, competence, and coping. Child Abus. Negl..

[32] Szanton S.L., Gill J.M. (2010). Facilitating resilience using a society-to-cells framework: A theory of nursing essentials applied to research and practice. Adv. Nurs. Sci..

[33] Feder A., Nestler E.J., Westphal M., Charney D.S. (2010). Psychobiological mechanisms of resilience to stress. Handbook of Adult Resilience.

[34] Farquhar J., Kamei R., Vidyarthi A. (2018). Strategies for enhancing medical student resilience: Student and faculty member perspectives. Int. J. Med. Educ..

[35] Ang W.H.D., Chew H.S.J., Dong J., Yi H., Mahendren R., Lau Y. (2022). Digital training for building resilience: Systematic review, meta-analysis, and meta-regression. Stress Health.

[36] Alvord M.K., Rich B.A., Berghorst L.H., Norcross J.C., VandenBos G.R., Freedheim D.K., Pole N. (2016). Resilience Interventions. APA Handbook of Clinical Psychology: Psychopathology and Health.

[37] Moore G., Audrey S., Barker M., Bond L., Bonell C., Cooper C., Hardeman W., Moore L., O’Cathain A., Tinati T. (2014). Process evaluation in complex public health intervention studies: The need for guidance. J. Epidemiol. Community Health.

[38] Moore G.F., Barker M., Bonell C., Hardeman W., O’Cathain A., Wight D., Baird J. (2015). Process evaluation of complex interventions: Medical Research Council guidance. BMJ.

[39] Carroll C., Patterson M., Wood S., Booth A., Rick J., Balain S. (2007). A conceptual framework for implementation fidelity. Implement. Sci..

[40] Grant A., Treweek S., Dreischulte T., Foy R., Guthrie B. (2013). Process evaluations for cluster-randomised trials of complex interventions: A proposed framework for design and reporting. Trials.

[41] Bonell C., Fletcher A., Morton M., Lorenc T., Moore L. (2012). Realist randomised controlled trials: A new approach to evaluating complex public health interventions. Soc. Sci. Med..

[42] Ungar M., Liebenberg L. (2013). Ethnocultural factors, resilience, and school engagement. Sch. Psychol. Int..

[43] Panter-Brick C., Eggerman M. (2012). Understanding culture, resilience, and mental health: The production of hope. The Social Ecology of Resilience.

[44] Seemiller C., Clayton J. (2019). Developing the strengths of generation Z college students. J. Coll. Character.

[45] Nicholas A.J. (2020). Preferred Learning Methods of Generation Z. https://digitalcommons.salve.edu/fac_staff_pub/74.

[46] Joyce S., Shand F., Tighe J., Laurent S.J., Bryant R.A., Harvey S.B. (2018). Road to resilience: A systematic review and meta-analysis of resilience training programmes and interventions. BMJ Open.

[47] Tong A., Sainsbury P., Craig J. (2007). Consolidated criteria for reporting qualitative research (COREQ): A 32-item checklist for interviews and focus groups. Int. J. Qual. Health Care.

[48] Craig P., Macintyre S., Michie S., Nazareth I., Petticrew M. (2008). Developing and evaluating complex interventions: The new Medical Research Council guidance. BMJ.

[49] Folkman S., Lazarus R.S. (1984). Stress, Appraisal, and Coping.

[50] Ang W.H.D., Shorey S., Lopez V., Chew H.S.J., Lau Y. (2021). Generation Z undergraduate students’ resilience during the COVID-19 pandemic: A qualitative study. Curr. Psychol..

[51] Sandelowski M. (1995). Sample Size in Qualitative Research. Res. Nurs. Health.

[52] Connor K.M., Davidson J.R. (2003). Development of a new resilience scale: The Connor-Davidson resilience scale (CD-RISC). Depress. Anxiety.

[53] Braun V., Clarke V. (2021). To saturate or not to saturate? Questioning data saturation as a useful concept for thematic analysis and sample-size rationales. Qual. Res. Sport Exerc. Health.

[54] Braun V., Clarke V., Cooper H., Camic P.M., Long D.L., Panter A.T., Rindskopf D., Sher K.J. (2012). Thematic Analysis. APA Handbook of Research Methods in Psychology, Vol. 2. Research Designs: Quantitative, Qualitative, Neuropsychological, and Biological.

[55] Glaser B.G. (1965). The constant comparative method of qualitative analysis. Soc. Probl..

[56] Krefting L. (1991). Rigor in qualitative research: The assessment of trustworthiness. Am. J. Occup. Ther..

[57] Joyce S., Shand F., Bryant R.A., Lal T.J., Harvey S.B. (2018). Mindfulness-based resilience training in the workplace: Pilot study of the internet-based Resilience@ Work (RAW) mindfulness program. J. Med. Internet Res..

[58] Abbott J.-A., Klein B., Hamilton C., Rosenthal A. (2009). The impact of online resilience training for sales managers on wellbeing and performance. Sens. A J. Mind Brain Cult..

[59] Kolb D.A. (2014). Experiential Learning: Experience as the Source of Learning and Development.

[60] Kolb D.A., Boyatzis R.E., Mainemelis C. (2014). Experiential learning theory: Previous research and new directions. Perspectives on Thinking, Learning, and Cognitive Styles.

[61] Cilliers E.J. (2017). The challenge of teaching generation Z. People: Int. J. Soc. Sci..

[62] Seemiller C., Grace M. (2016). Generation Z Goes to College.

[63] Bandura A., Walters R.H. (1977). Social Learning Theory.

[64] Lowder L. (2013). Engaging Students through Online Discussion Forums; Expectations to Promote Social Learning. E-Learn: World Conference on E-Learning in Corporate, Government, Healthcare, and Higher Education, Las Vegas, NV, USA, 21 October 2013.

[65] Chen B., Chang Y., Ouyang F., Zhou W. (2018). Fostering student engagement in online discussion through social learning analytics. Internet High. Educ..

[66] Muthuprasad T., Aiswarya S., Aditya K.S., Jha G.K. (2021). Students’ perception and preference for online education in India during COVID-19 pandemic. Soc. Sci. Humanit. Open.

[67] Wanner T., Palmer E. (2015). Personalising learning: Exploring student and teacher perceptions about flexible learning and assessment in a flipped university course. Comput. Educ..

[68] Evans D.J., Zeun P., Stanier R.A. (2014). Motivating student learning using a formative assessment journey. J. Anat..

[69] Rüth M., Breuer J., Zimmermann D., Kaspar K. (2021). The effects of different feedback types on learning with mobile quiz apps. Front. Psychol..

[70] McKay M., Wood J.C., Brantley J. (2019). The Dialectical Behavior Therapy Skills Workbook: Practical DBT Exercises for Learning Mindfulness, Interpersonal Effectiveness, Emotion Regulation, and Distress Tolerance.

[71] Jha A.P., Morrison A.B., Parker S.C., Stanley E.A. (2017). Practice is protective: Mindfulness training promotes cognitive resilience in high-stress cohorts. Mindfulness.

[72] Morgan L.P., Graham J.R., Hayes-Skelton S.A., Orsillo S.M., Roemer L. (2014). Relationships between amount of post-intervention mindfulness practice and follow-up outcome variables in an acceptance-based behavior therapy for Generalized Anxiety Disorder: The importance of informal practice. J. Context. Behav. Sci..

[73] Kim H.-J., Lee J.-M., Rha J.-Y. (2017). Understanding the role of user resistance on mobile learning usage among university students. Comput. Educ..

[74] Olson J.S., Kenahan R. (2021). “An Overwhelming Cloud of Inertia”: Evaluating the Impact of Course Design Changes Following the COVID-19 Pandemic. Online Learn..

[75] Barcelona A.B. (2017). An assessment of the non-graded system based on learners’ learning satisfaction, beahavior, and outcomes. People Int. J. Sci..

[76] McMorran C., Ragupathi K. (2020). The promise and pitfalls of gradeless learning: Responses to an alternative approach to grading. J. Furth. High. Educ..

[77] Rasheed F. (2007). Factors impeding implementation of web-based distance learning. AACE Rev..

[78] Alkhasawnh S., Alqahtani M.A.M. (2019). Fostering students’ self-regulated learning through using a learning management system to enhance academic outcomes at the University of Bisha. TEM J..

[79] Lonn S., Teasley S.D. (2009). Saving time or innovating practice: Investigating perceptions and uses of Learning Management Systems. Comput. Educ..

[80] Przybylko G., Morton D.P., Renfrew M.E. (2021). Addressing the COVID-19 mental health crisis: A perspective on using interdisciplinary universal interventions. Front. Psychol..

[81] de Paiva Azevedo J., Delaney H., Epperson M., Jbeili C., Jensen S., McGrail C., Weaver H., Baglione A., Barnes L.E. Gamification of ehealth interventions to increase user engagement and reduce attrition. Proceedings of the 2019 Systems and Information Engineering Design Symposium (SIEDS).

[82] Smaldone R.A., Thompson C.M., Evans M., Voit W. (2017). Teaching science through video games. Nat. Chem..

[83] Looyestyn J., Kernot J., Boshoff K., Ryan J., Edney S., Maher C. (2017). Does gamification increase engagement with online programs? A systematic review. PLoS ONE.

[84] Poondej C., Lerdpornkulrat T. (2019). Gamification in e-learning: A Moodle implementation and its effect on student engagement and performance. Interact. Technol. Smart Educ..

[85] Vizcaya-Moreno M.F., Pérez-Cañaveras R.M. (2020). Social media used and teaching methods preferred by generation z students in the nursing clinical learning environment: A cross-sectional research study. Int. J. Environ. Res. Public Health.

[86] Hew K.F., Cheung W.S. (2014). Students’ and instructors’ use of massive open online courses (MOOCs): Motivations and challenges. Educ. Res. Rev..

[87] Nguyen T. (2015). The effectiveness of online learning: Beyond no significant difference and future horizons. MERLOT J. Online Learn. Teach..

[88] Buss B., Krautter M., Moltner A., Weyrich P., Werner A., Junger J., Nikendei C. (2012). Can the “Assessment Drives Learning” effect be detected in clinical skills training?-Implications for curriculum design and resource planning. GMS Z Für Med. Ausbild..

[89] Murray D.J., Boulet J.R. (2018). Anesthesiology Board Certification Changes: A Real-time Example of “Assessment Drives Learning”. Anesthesiology.

[90] Woodyatt C.R., Finneran C.A., Stephenson R. (2016). In-person versus online focus group discussions: A comparative analysis of data quality. Qual. Health Res..

[91] Frost J., Vermeulen I.E., Beekers N. (2014). Anonymity versus privacy: Selective information sharing in online cancer communities. J. Med. Internet Res..

